# β-Hydroxyisovaleryl-shikonin induces human cervical cancer cell apoptosis via PI3K/AKT/mTOR signaling

**DOI:** 10.3892/ol.2021.12456

**Published:** 2021-01-08

**Authors:** Dan Lu, Jing Qian, Wei Li, Qianqian Feng, Shu Pan, Siquan Zhang

Oncol Lett 10: 3434-3442, 2015; DOI: 10.3892/ol.2015.3769

Subsequently to the publication of the above paper, an interested reader drew to the authors’ attention that, on p. 3437, the data shown in Fig. 2B and C were strikingly similar. The authors have re-examined their data, and realized that the data correctly shown for Fig. 2B was reused in Fig. 2C. However, the authors were able to locate the original data pertaining to Fig. 2C, and the corrected version of Fig. 2 is shown opposite.

The authors regret the error that was made in the preparation of the published figure, and confirm that this error did not affect the conclusions reported in the study. The authors are grateful to the editor of *Oncology Letters* for allowing them the opportunity to publish a Corrigendum, and all the authors agree to this Corrigendum. Furthermore, they apologize to the readership for any inconvenience caused.

## Figures and Tables

**Figure 2.**
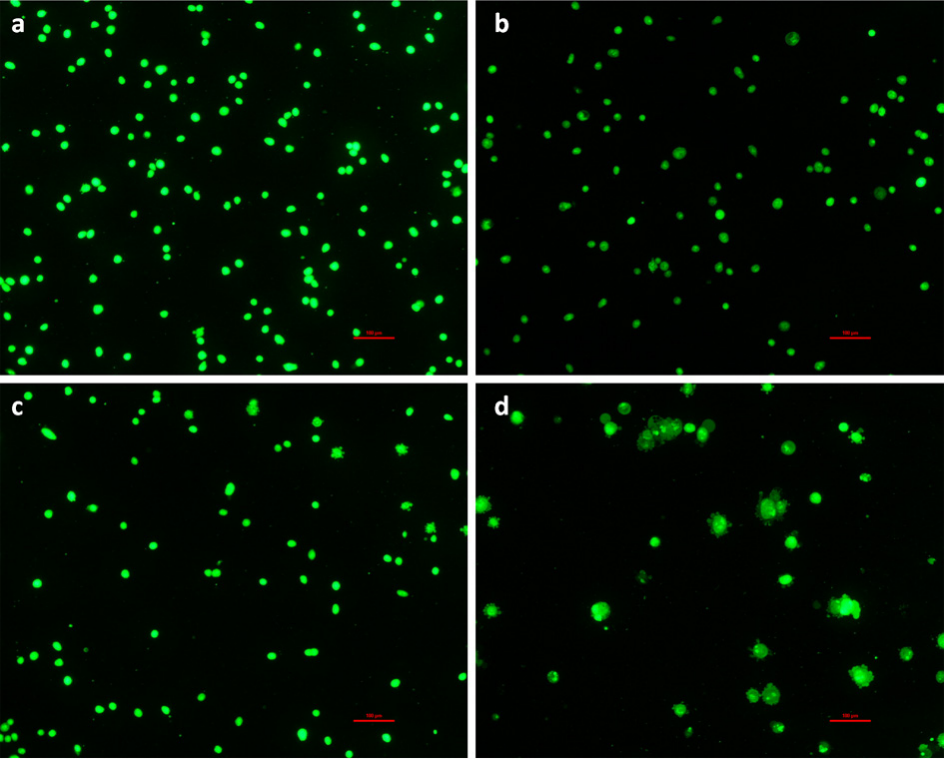
Acridine orange/ethidium bromide staining for morphological changes in apoptotic HeLa cells at 24 h in the (A) control group and cells treated with (B) 1 µM β-HIVS, (C) 5 µM β-HIVS and (D) 10 µM β-HIVS. Magnification, ×100. β-HIVS, β-hydroxyisovaleryl-shikonin.

